# Spatial-Temporal Pattern and Evolution Trend of the Cultivated Land Use Eco-Efficiency in the National Pilot Zone for Ecological Conservation in China

**DOI:** 10.3390/ijerph19010111

**Published:** 2021-12-23

**Authors:** Zhenggen Fan, Chao Deng, Yuqi Fan, Puwei Zhang, Hua Lu

**Affiliations:** 1 College of City Construction, Jiangxi Normal University, Nanchang 330022, China; fanzg@jxnu.edu.cn (Z.F.); 202040100793@jxnu.edu.cn (C.D.); zhang.p.w@jxnu.edu.cn (P.Z.); 2 Institute of Ecological Civilization, Jiangxi University of Finance and Economics, Nanchang 330013, China

**Keywords:** land use, CLUE, temporal and spatial evolution, spatial spillover, national pilot zone for ecological conservation in China

## Abstract

The cultivated land use eco-efficiency (CLUE) is an important indicator to evaluate ecological civilization construction in China. Research on the spatial-temporal pattern and evolution trend of the CLUE can help to assess the level of ecological civilization construction and reveal associated demonstration and driving effects on surrounding areas. Based on the perspective of the CLUE, this paper obtains cultivated land use data pertaining to National Pilot Zones for Ecological Conservation in China and neighboring provinces from 2008 to 2018. In this study, the SBM-undesirable, Moran’s I, and Markov chain models are adopted to quantitatively measure and analyze the CLUE and its temporal and spatial patterns and evolution trend. The research results indicate that the CLUE in the whole study area exhibited the characteristics of one growth, two stable, and two decline stages, with a positive spatial autocorrelation that increased year by year, and a spatial spillover effect was observed. Geographical spatial patterns and spatial spillover effects played a major role in the evolution of the CLUE, and there occurred a higher probability of improvement in the vicinity of cities with high CLUE values. In the future, practical construction experience should be disseminated at the provincial level, and policies and measures should be formulated according to local conditions. In addition, a linkage model between prefecture-level cities should be developed at the municipal level to fully manifest the positive spatial spillover effect. Moreover, we should thoroughly evaluate the risk associated with CLUE transition from high to low levels and establish a low-level early warning mechanism.

## 1. Introduction

With increasing social and economic development levels, cultivated land notably functions as the basic means of agricultural production, provides ecological products, and plays a significant role in ensuring both national food and ecological security. However, with the rapid advancement of industrialization and urbanization in China, cultivated land also faces difficulties such as sharp reductions in quantity and quality, and idle abandonment. On the premise of ensuring national food security, this has led to changes in the cultivated land input and production structure, and an agricultural production mode dominated by petrol-agriculture has gradually been established. The accompanying changes in cultivated land use intensity have significantly undermined the integrity of biodiversity [1], and seriously threatened the quality of cultivated land habitat [2], accordingly resulting in food security problems in China [3]. According to the Second National Pollution Source Census released by China in 2020, in 2017, the ammonia nitrogen emissions of the planting industry in China reached 83,000 tons, the total phosphorus emissions reached 76,200 tons, the use of plastic films reached 1,419,300 tons, and the accumulated residue reached 1,184,800 tons over time. The anti-ecological effect of cultivated land utilization has gradually accumulated, thereby seriously restricting the green development of cultivated land utilization in China. Based on these aspects, the 18th Communist Party of China (CPC) National Congress report clearly proposed the five-in-one overall layout accounting for ecological civilization construction, which requires comprehensive consideration of regional economic development and ecological civilization construction to promote coordinated development. Moreover, to further promote the implementation of ecological civilization construction at the national level, the Central Committee of the CPC further selected and deployed Fujian, Jiangxi, and Guizhou provinces with a good ecological foundation and a high resource and environmental carrying capacity as the first batch of National Pilot Zones for Ecological Conservation in China to take the lead in exploration and provide model experience for ecological civilization construction in other regions. Agriculture constitutes an essential part of promoting regional ecological civilization construction, and cultivated land is the primary material carrier and production factor. Therefore, optimization of the input-output structure of cultivated land use and improvement of the cultivated land use efficiency have become critical paths to realize regional agricultural ecological civilization construction. Therefore, within this context, methods to enhance ecological use of cultivated land and ensure the coupling and coordination between cultivated land use and ecological environment have become critical aspects to promote ecological civilization construction.

The concept of eco-efficiency was proposed in 1990. It is an important indicator for measuring the construction of regional ecological civilization. It refers to the ratio of economic growth to environmental impact and emphasizes the coordinated development of economic growth and ecological environment [4]. In 1996, the World Business Council for Sustainable Development (WBCSD) further deepened and expanded the concept, and proposed that in the process of resource consumption, it is not only required to meet the basic needs of human society, but also to ensure that the ecological environmental impact is consistent with the environmental carrying capacity of the Earth [5]. This concept takes into account both social and economic development and resource environmental protection, which effectively solves the problem of how to quantify the two at the same level, and so it has been gradually expanded and applied by various institutions and scholars in different fields. The existing studies are mainly focused on the basic theory of eco-efficiency [6,7,8] and practical applications in different fields. The application fields cover agriculture [9,10], industry [11,12], manufacturing industry, etc. [13,14], and the application areas cover cities [15,16], regions [17,18], and countries [19]. Among them, the practical applications involved in the field of land use mainly focus on land management, cultivated land compensation, intensive land use, land use zoning, land use transformation, etc. [20,21,22,23,24].

The eco-efficiency of land use, as an important indicator to quantify the construction of ecological civilization in the field of land use, accurately reflects the degree of coordination between regional land use and the ecological environment, and accordingly has been widely applied and implemented by scholars. The existing studies mainly focus on the design of methods for measuring the eco-efficiency of land use, and the analysis of the spatial and temporal characteristics of regional eco-efficiency of land use and its influencing factors. The measurement methods mainly include ecological footprint [21,25], principal component analysis (PCA) [12,26] and data envelopment analysis (DEA), etc. [27,28,29]. Among these methods, the DEA-SBM model derived on the basis of DEA does not need to set the specific form and estimation parameters of the model in advance, and can effectively solve the slack problem of input and output variables, and thus has become the mainstream model for measuring the eco-efficiency of land use in the current academic circles. As regards the evolution of temporal and spatial characteristics, scholars mainly analyzed the temporal and spatial variation in eco-efficiency of land use [30,31], and summarized and elaborated the temporal and spatial evolution rules of eco-efficiency of land use in a specific study area [32,33]. In terms of the analysis of influencing factors, existing studies generally incorporate socio-economic development situations, the marketization level, the industrial development state, and the ecological input level into the analysis system of factors influencing the eco-efficiency of land use [33,34,35,36,37]. Specifically, the economic development level, industrial agglomeration and openness, and ecological input are considered to exert a positive effect on promoting the eco-efficiency of land use [33,34,35,36], while the land marketization level, urban-rural income gap, and ecological pressure are considered to play a negative hindering role in the eco-efficiency of land use [33,36,37].

As one of the most fundamental elements of agricultural production, cultivated land not only has the social and economic service functions of producing food to ensure the regional food security, but also has ecological service functions such as conserving water and soil resources, regulating climate, and protecting biodiversity [38,39]. Therefore, to strengthen the multi-functional value of cultivated land, quantify the environmental efficiency loss caused by cultivated land use, and realize the sustainable use of cultivated land, some scholars gradually shifted their research perspectives on the eco-efficiency of land use to the cultivated land use eco-efficiency (CLUE). At present, the research achievements of the CLUE are mainly concentrated on the measurement of application methods, including two categories: the ecological footprint method [25] and the DEA method [27]; at the same time, some scholars analyzed factors influencing the CLUE [34].

The above relevant research laid a solid theoretical foundation for this paper, but there is still room for further research in the following two aspects: (1) cultivated land use is an important activity in the process of agricultural production. Exploring the CLUE is highly important to promote agricultural ecological civilization construction. However, there exists relatively little research in this field in current academic circles, and it is difficult to establish a policy system, especially given the current measures aimed at vigorously promoting the experimental area of ecological civilization in China. The lack of relevant research on National Pilot Zones for Ecological Conservation in China makes it challenging to achieve a demonstration effect at the national level, which readily limits the effectiveness of these zones. (2) Existing research has only measured the CLUE within a certain region to analyze temporal and spatial evolution patterns, but the spillover effect has not been sufficiently explored. However, according to the first law of geography, each object in a geographical space exhibits a specific spatial autocorrelation. Therefore, in CLUE exploration research, we should consider the inherent spillover phenomenon. Based on this consideration, we can more accurately describe the temporal and spatial evolution rules of the CLUE.

Based on the above analysis of existing relevant research, the aims of this paper can be summarized as follows: (1) first, this paper aims to construct a CLUE evaluation index system from an ecological perspective. Taking the first batch of National Pilot Zones for Ecological Conservation in China and surrounding related prefecture-level cities as the research area, it measures the CLUE from 2008 to 2018 using the SBM-undesirable model, and analyzes the spatial-temporal evolution characteristics of the CLUE. (2) Then, traditional and spatial Markov chain models are constructed to empirically examine the evolution trend and the spatial spillover effect of the CLUE within the study area, and analyze the formation process of regional spatial agglomeration phenomena and the spatial spillover effect. (3) Finally, the fundamental strategies for low-carbon, low-pollution and efficient cultivated land use are put forward, and development ideas are assessed for agricultural ecological construction in National Pilot Zones for Ecological Conservation in China to provide policy recommendations for efficient cultivated land use and agricultural ecological construction in these zones.

## 2. Research Methods and Data Sources

### 2.1. Overview of the Study Area

**Figure 1:**
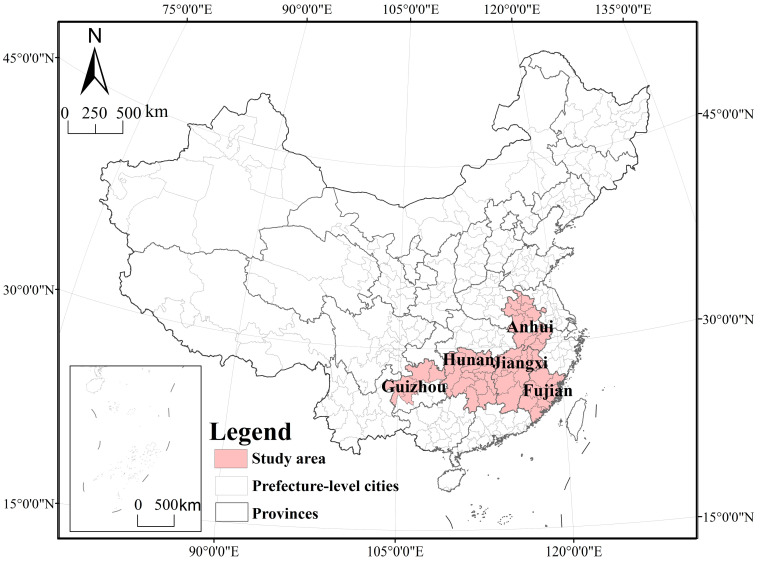
The geographical location of study area.

Jiangxi, Fujian, and Guizhou comprise the first batch of National Pilot Zones for Ecological Conservation in China, with an excellent ecological environment, but the ecological advantage does not directly match the economic advantage. The economic development process of areas with a certain ecological advantage has occurred at a disadvantage for a long time [40], which is quite evident in the agricultural economy. Moreover, with increasing agricultural mechanization levels in China, regional carbon emissions have continuously risen. In addition, farmers use land intensively, but cultivated land is lightly maintained in the process of cultivated land utilization, while large amounts of chemical fertilizers and pesticides are applied, resulting in increasing pressure on regional resources and the environment and worsening environmental problems. In addition, ecological civilization construction is facing challenges. Therefore, in response to the call to reduce the application of chemical fertilizers and pesticides, decrease the discharge of harmless agricultural solid waste and improve green ecological agriculture, as proposed by the state in the Implementation Plan for National Ecological Civilization Pilot Zones, we must correctly understand the coupling and coordination of economic development and ecological protection in cultivated land utilization and strive to realize the unification and optimization of ecological, economic and social benefits, thereby promoting the construction of National Pilot Zones for Ecological Conservation in China. Based on these considerations, this paper selects 57 prefecture-level cities in the first batch of National Pilot Zones for Ecological Conservation in China (Jiangxi, Fujian, and Guizhou), in addition to Anhui and Hunan provinces (Figure 1), as the research object, studies the temporal and spatial patterns and evolution trend of the CLUE from the perspective of comparative analysis and provides theoretical support to improve the construction and leading role of National Pilot Zones for Ecological Conservation in China.

### 2.2. Research Methods

#### 2.2.1. SBM-Undesirable Model

The SBM-undesirable model can solve the problem of efficiency measurement, including unexpected outputs, and can avoid result deviations due to radial and angular problems. This model is a scientific evaluation method established through the continuous improvement of the traditional DEA model according to practical experience. It has been widely used in efficiency evaluation in various research fields [41,42,43], and it is also a mainstream measurement method for land use efficiency evaluation in recent years [44,45,46]. Because CLUE calculation results can differ between variable return to scale (VRS) and constant return to scale (CRS), combined with the research of Zhou P. et al. [47,48,49,50], the SBM-undesirable model based on the VRS is adopted, which can better reflect the essence of the CLUE. Its basic principles are expressed as follows:

In the process of cultivated land use, it is assumed that there are n decision-making units, all corresponding to m input indicators $x_{i0}$, a expected output indicators $y_{r0}^{n}$ and b unexpected output indicators $y_{h0}^{n}$. Then, matrices X,

$$Y^{e}$$,

and $Y^{n}$ can be defined as ($x_{1}$, $x_{2}$, …, $x_{n}$)∈$R^{m\times n}$, ($y_{1}^{e}$, $y_{2}^{e}$, …, $y_{n}^{e}$)∈$R^{a\times n}$, ($y_{1}^{n}$, $y_{2}^{n}$, …, $y_{n}^{n}$)∈ $R^{b\times n}$, respectively. Moreover, assuming that X, $Y^{e}$, and $Y^{n}$ are greater than zero, the production possibility set can be defined as $P_{ij}(N)$, and the SBM-undesirable model can be expressed as:

(1) $$\rho^{*}=\min\frac{1-\frac{1}{m}\sum_{i=1}^{m}\frac{D_{i}^{-}}{x_{i0}}}{1+\frac{1}{a+b}(\sum_{r=1}^{a}\frac{D_{r}^{e}}{y_{r0}^{e}}+\sum_{h=1}^{b}\frac{D_{h}^{n}}{y_{h0}^{n}})}$$

$s.t.x_{0}=X\lambda +D^{-},y_{0}^{e}=Y^{e}\lambda -D^{e},$ and $y_{0}^{n}=Y^{n}\lambda +D^{n}$.

$D^{-}\geq 0,D^{e}\geq 0,D^{n}\geq 0,$ and λ≥0.

In Equation (1), $\rho^{*}$ denotes the CLUE value in each region within the study area, and the value ranges from 0 to 1. When the $\rho^{*}$ value is 1, this indicates that the process is entirely effective. For $\rho^{*}$ < 1, efficiency loss occurs, and there exists room for further optimization. The number of inputs and the expected and unexpected outputs are denoted as m, a, and b, respectively, and the corresponding slack variables are denoted as $D^{-}$, $D^{e}$, and $D^{n}$, respectively. Furthermore, the corresponding input-output values are denoted as $x_{i0}$, $y_{r0}^{n}$, and $y_{h0}^{n}$, respectively, and λ denotes the weight vector.

#### 2.2.2. Spatial Autocorrelation Model

Spatial autocorrelation analysis is an effective method to describe spatial correlation and spatial heterogeneity by panel data. Spatial geographical relations are integrated into data analysis through global Moran’s I [51,52,53]. The global Moran’s index (Moran’s I) reflects the spatial correlation characteristics of the CLUE from a global perspective, and the model is expressed in Equation (2):

(2) $$I=\frac{\sigma^{2}\sum_{i=1}^{n}\sum_{j=1}^{n}w_{ij}(x_{i}-\bar{x})(x_{j}-\bar{x})}{\sigma^{2}\sum_{i=1}^{n}\sum_{j=1}^{n}w_{ij}}$$

In Equation (2), I is Moran’s I, n is the total number of evaluation units in the study area, $x_{i}$ and $x_{j}$ are the attribute values of evaluation units i and j, respectively (i=j), $\bar{x}$ is the average CLUE value of the evaluation unit, and $\sigma^{2}$ is the sample variance. For I>0, a positive spatial correlation exists in terms of the CLUE. For I<0, a negative spatial correlation exists. The magnitude of the positive or negative I values reflects the degree of spatial positive or negative correlation, respectively, and $w_{ij}$ is the spatial weight matrix, which reflects the spatial adjacency relationship between the evaluation units. The data in this paper are based on panel data pertaining to the study area, and the Queen adjacency matrix based on GeoDa is adopted. Then, a certain criterion is applied to construct the spatial weight matrix.

The global spatial autocorrelation reflects the average correlation and the different degrees of the CLUE in the overall space but cannot reflect the specific characteristics of local spatial aggregation or differentiation. Therefore, to implement the local spatial autocorrelation method for analysis purposes, this paper adopts local Moran’s I, and the calculation equation is as follows:

(3) $$I_{i}=\frac{\left(x_{i}-\bar{x}\right)}{\sigma^{2}}\sum_{j=1}^{n}w_{ij}\left(x_{j}-\bar{x}\right)$$

In Equation (3), $I_{i}$ is the local Moran’s I of evaluation unit i. Positive or negative $I_{i}$ values correspond to adjacent areas with similar or different CLUE values, respectively. The absolute value of $I_{i}$ reflects the degree of spatial proximity.

#### 2.2.3. Markov Chain Model

The Markov chain model determines the change trend of each state of objects through the initial probability of different states and the transition probability between states. In Environmental Science, it is applied to analyze the spatial-temporal dynamic evolution characteristics of things [54,55]. In this paper, traditional and spatial Markov chain models are adopted for analysis.

According to the state type of the CLUE, the traditional Markov chain model can construct an N×N-order Markov probability transfer matrix to analyze the temporal evolution characteristics of regional CLUE values. Assuming that $P_{ij}$ is the transition probability of the CLUE of a given unit in the study area from state $E_{i}$ to state $E_{j}$ from year t to year t + 1, the value can be estimated with Equation (4), as follows:

(4) $$P_{ij}(E_{i}\to E_{j})=\frac{n_{ij}}{n_{i}}$$

In Equation (4), $n_{ij}$ denotes the total number of regional units as the state type of the CLUE transitions from $E_{i}$ to $E_{j}$, and $n_{i}$ denotes the number of regional units with $E_{i}$ occurring at the i level.

The spatial Markov chain model combines the traditional Markov chain with the concept of the spatial lag, which can explore the mechanism of the spatial spillover effect in the temporal and spatial transfer processes of the CLUE and can be applied to analyze the possibility of CLUE transfer against different geospatial backgrounds to explore the internal relationship between the evolution process of the CLUE and the regional background. Under the condition of spatial lag $N_{i}$, the traditional N×N-order Markov probability transfer matrix is decomposed into an N×N×N-order probability transfer matrix. $P_{ij}(N)$ indicates that under the condition of spatial lag $N_{i}$, the possibility of CLUE transfer shifts from type $E_{i}$ into type $E_{j}$.

### 2.3. Index System Construction and Data Sources

Referring to relevant research results [37,56], the evaluation index system of the CLUE constructed from an ecological perspective should cover three aspects, namely, the input, expected output, and unexpected output, of the four systems of resources, economy, nature, and society. The constructed index system of the CLUE is provided in Table 1.

In terms of the input index, the actual sowing area of crops (1000 hm^2^), number of employees (10,000 persons), net amount of pesticide application (t), and net amount of chemical fertilizer application (t) were selected as representative indicators. In terms of the expected output index, the total grain output (t) and planting output value (10,000 yuan) were selected as representative indicators. In terms of the unexpected output index, the difference between the total carbon emissions (t) and total carbon absorption (t) was selected as a representative indicator.

The basic input and output data required to measure the CLUE in the study area were retrieved from the *China Statistical Yearbook 2009–2020*, *China Rural Statistical Yearbook 2009–2020*, provincial and municipal statistical yearbooks, and statistical bulletins. The acquired carbon emission data were related to chemical fertilizers, pesticides, and agricultural films. These data were obtained by multiplying and summarizing basic data, such as mechanized operation and cultivated land plowing data. The carbon emission coefficients of the various carbon sources were determined based on the carbon emission model and calculation coefficients of West and Marland et al. [57,58]. The carbon absorption coefficient of cultivated land was set to 0.0070 t/hm, as reported by He Yong et al. [59]. Correlation measurement coefficients were obtained from Liang Liu Tao and Feng Yonggang et al. [60,61].

## 3. Analysis of the Empirical Results

### 3.1. Temporal Dynamic Evolution Characteristics of the CLUE

**Figure 2:**
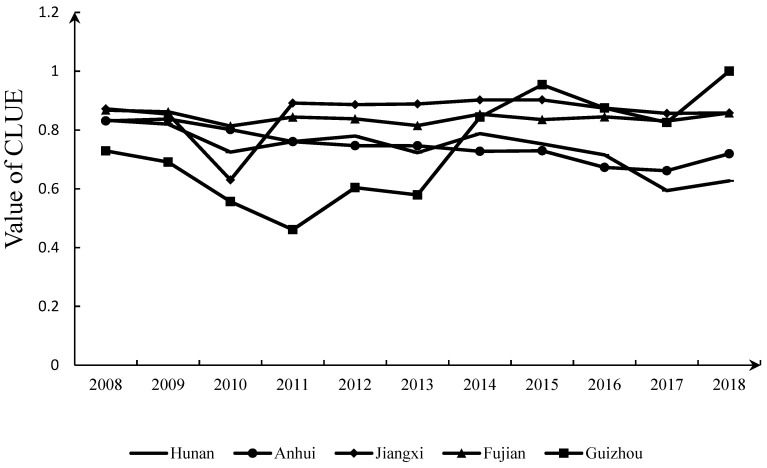
Change trend of the CLUE in the study region from 2008 to 2018.

The CLUE was measured with DEA-SOLVER PRO13 software. According to the overall observations, the CLUE in the study area was notably different from 2008 to 2018, thereby exhibiting the characteristics of one growth, two stable, and two decline stages (Figure 2).

The growth stage suggests that the CLUE in Guizhou Province experiences a growth trend, with an average annual growth rate of 3.21%, which occupies the leading position in the region (Figure 2). This suggests that ecological civilization construction in Guizhou Province achieved remarkable results in cultivated land utilization. From 2008 to 2018, the average input of pesticides and chemical fertilizers in Guizhou Province reached 187.42 t/1000 hm^2^, the lowest in the whole region, 27.3% lower than that in Jiangxi Province, which ranked second-lowest. In terms of the land average net carbon emissions, the land average net carbon emissions in Guizhou Province decreased from 238.87 T/1000 hm^2^ in 2008 to 1869.18 T/1000 hm^2^ in 2018. These land average net carbon emissions were the lowest in the study area, and Guizhou Province was the only province indicating a decline in emissions. In terms of the average annual growth rate of the agricultural output value, Guizhou Province attained a rate of 15.79%, while Jiangxi, Fujian, Anhui, and Hunan attained rates of 7.82%, 7.57%, 1.49%, and 0.24%, respectively, of which the annual average growth rate in Guizhou Province was much higher than that in the other provinces within the study area. Based on the above three groups of data, it is observed that although the initial average agricultural output value in Guizhou Province was the lowest, the expected output value growth rate was the highest, and the average pesticide and chemical fertilizer input and average net carbon emissions remained the lowest. Moreover, these findings are the main reasons why the CLUE in Guizhou Province has taken the lead in the study area in recent years.

The two stable stages and two decline stages suggest that the CLUE exhibited the characteristics of high and stable fluctuations in Jiangxi and Fujian and an overall downward trend in Hunan and Anhui, respectively (Figure 2). The average CLUE values in Jiangxi and Fujian were 0.856 and 0.842, respectively, ranking as the top two highest values in the study area, but the fluctuation range was smaller than 2%. The CLUE in Hunan and Anhui revealed a downward trend. In 2018, the CLUE in these two provinces reached 0.62 and 0.719, declines of 23.8% and 13.4%, respectively, compared to 2008. The reason why the CLUE in Jiangxi and Fujian remained high with stable fluctuations could be that the ecological basis of cultivated land use in these two provinces is good. The average grain production in Jiangxi and Fujian provinces from 2008 to 2018 reached 4113.6 and 2894.3 t/1000 hm^2^, respectively, higher than the average grain production values of 2473.8 t/1000 hm^2^ in Hunan and 2136.4 t/1000 hm^2^ in Anhui Province. Moreover, the average annual net carbon emissions in these two provinces reached 1,955,700 and 1,642,300 tons, respectively, lower than those in Hunan (3,379,500 tons) and Anhui (3,942,800 tons). Compared to the data for Jiangxi and Fujian provinces within the ecological civilization construction experimental area in 2018, Anhui and Hunan provinces exhibited room for improvement by approximately 19% and 36%, respectively, in the CLUE.

### 3.2. Spatial Evolution Characteristics of the CLUE

#### 3.2.1. Overall Spatial Evolution Characteristics

Table 2 indicates that the global spatial autocorrelation of the CLUE within the whole region is increasing, and the spatial correlation between adjacent regions is increasingly intensifying. From 2008 to 2018, global Moran’s I value of the CLUE increased from 0.136 to 0.323, and the significance test result increased from 5% to 1%, indicating a fluctuating upward trend. This suggests that there occurs a significant positive spatial autocorrelation in regard to the CLUE in the study area.

#### 3.2.2. Local Evolution Characteristics of the CLUE

**Figure 3:**
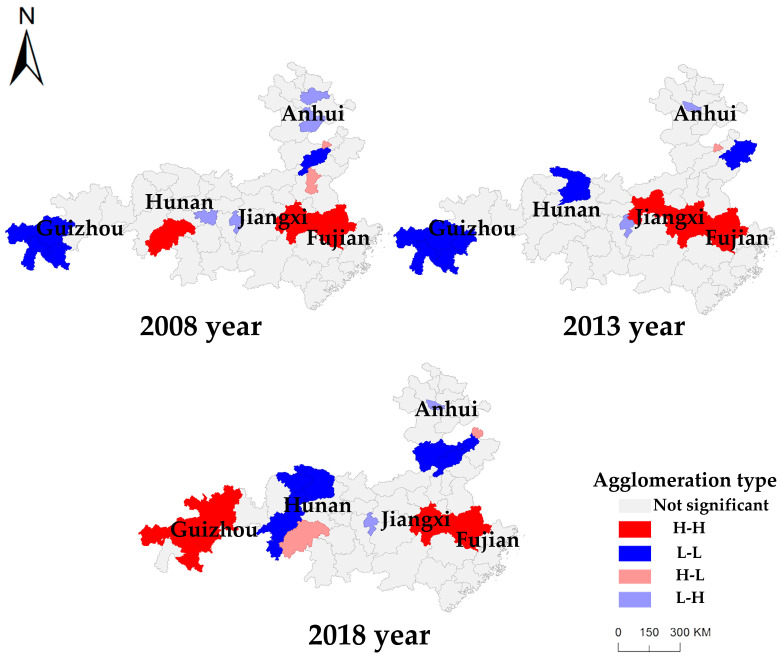
LISA cluster map of the CLUE in the study area from 2008 to 2018.

To further analyze the specific spatial agglomeration characteristics of the CLUE, according to the calculation results of local Moran’s I at the 10% significance level, a Local Indicators of Spatial Association (LISA) cluster diagram of the CLUE in the study area for 2008, 2013, and 2018 was generated (Figure 3). As shown in the figure, the CLUE in the study area exhibited significant high-high (H-H) and low-low (L-L) agglomeration phenomena within the geographical space encompassing prefecture-level cities, which became increasingly significant over time.

In 2008, the H-H and L-L agglomeration areas of the CLUE indicated the single-core agglomeration phenomenon. An H-H single-core aggregation area was located at the junction of Jiangxi and Fujian provinces in the National Pilot Zone for Ecological Conservation in China, comprising Fuzhou and Nanping. An L-L single-core aggregation area was located in western Guizhou Province, comprising Bijie, Liupanshui, and Anshun. The degree of single-core agglomeration was low in 2008.

In 2013, the H-H and L-L agglomeration areas of the CLUE revealed an increased scale of single-core agglomeration. An H-H single-core agglomeration area was still located at the junction of Jiangxi and Fujian provinces, comprising Yingtan, Yichun, Fuzhou, and Nanping. An L-L single-core agglomeration area was located in the west of Guizhou Province and comprised Guiyang, Bijie, Liupanshui, and Anshun. Compared to 2008, the geographical location of the H-H and L-L single-core agglomeration areas did not shift. According to the number of cities, the agglomeration scale expanded by 100% and 33.3%, respectively, and the positive spatial spillover effect was notable.

In 2018, the H-H and L-L agglomeration areas of the CLUE demonstrated the double-core agglomeration phenomenon. H-H dual-core agglomeration areas were located at the junction of Jiangxi and Fujian provinces and western Guizhou. The agglomeration area in western Guizhou Province represented a new agglomeration area. This agglomeration area comprised Guiyang, Zunyi, Bijie, and Anshun, exhibiting the characteristics of east–west H-H and dual-core agglomeration encompassing six cities. The L-L aggregation area shifted, and a new L-L dual-core aggregation area comprising seven cities was formed in Hunan and Anhui.

In summary, from 2008 to 2018, the spatial agglomeration of the CLUE in the study area exhibited the characteristics of agglomeration core-based expansion and transfer. In terms of agglomeration core-based expansion, a development trend was observed from two to four cores. In terms of agglomeration core-based transfer, the L-L agglomeration phenomenon in western Guizhou evolved into an H-H agglomeration phenomenon. The observed agglomeration phenomena indicated that there occurred a spatial spillover effect in the study area. In particular, when an adjacent area was observed with a high (low) level of the CLUE, the target area was more likely to become an area with a high (low) CLUE level. As of 2018, H-H aggregation areas of the CLUE were distributed among Fujian, Jiangxi, and Guizhou provinces, indicating that the positive spatial spillover effect of the National Pilot Zone for Ecological Conservation in China was notable. However, the effect was largely distributed within the National Pilot Zone for Ecological Conservation in China, and the driving effect on the surrounding provinces and cities of the National Pilot Zone for Ecological Conservation in China was not notable.

### 3.3. Markov Chain Analysis of the CLUE in the Study Area

#### 3.3.1. Traditional Markov Chain Analysis

According to the quantile division method, thereby adopting the first, second, and third quantiles as boundaries, the 57 prefecture-level cities in the study area from 2008 to 2018 were divided into four adjacent but nonintersecting state spaces with low, medium-low, medium-high, and high efficiency values according to the difference in the CLUE, denoted as levels Ⅰ, Ⅱ, Ⅲ and Ⅳ, respectively. The probability transition matrix of the traditional Markov chain analysis method was thus obtained (Table 3).

The CLUE in the whole region generally exhibited a consistent trend, and the convergence phenomenon was observed in regard to the extreme value. It was difficult to achieve a significant improvement over the short term, and there existed a certain transfer risk of to the medium-low state.

In terms of state maintenance, the probability along the diagonal at levels Ⅰ, Ⅱ and Ⅳ of the state space was significantly higher than that along the nondiagonal. Notably, the transformation probability of the CLUE at the same level was much higher than that between the different levels, in which the minimum value reached 0.647 and the maximum value reached 0.832. Under the above conditions, the CLUE attained a probability of at least 64.7% in the future development process and remained at the same level.

In terms of extreme value convergence, Table 3 demonstrates that the probability values of maintaining the current CLUE level along the diagonal followed the order of P_IV-IV_ (0.855) > P_I-I_ (0.832) > P_II-II_ (0.674) > P_III-III_ (0.365), and the probability values at both ends of the diagonal were significantly higher than the median value, indicating that the CLUE values were characterized by H-H and L-L agglomeration patterns, i.e., the core convergence phenomenon occurred.

In terms of efficiency improvement, except for P_III-II_ (0.385), the state transition probability along the nondiagonal was significantly lower than that along the diagonal, of which the maximum value reached 0.192 and the minimum value reached 0.007, indicating that it is difficult to greatly and rapidly improve the CLUE within a short timeframe. Long-term and effective ecological civilization construction is thus needed.

In terms of risk prediction, the CLUE could indicate a certain transfer risk from medium-high to medium-low efficiency values over the short term. The probability of transferring from type Ⅲ to type Ⅱ was significantly higher than that of transferring from type Ⅲ to types Ⅳ and Ⅰ (P_III-II_ (0.385) > P_III-III_ (0.365) > P_III-IV_ (0.192) > P_III-I_ (0.058)). These areas are more likely to fall into the low eco-efficiency trap.

#### 3.3.2. Spatial Markov Chain Analysis

Figure 3 shows that the spatial pattern of the CLUE in the study area exhibits significant spatial agglomeration characteristics. Therefore, a spatial lag was incorporated into the traditional Markov chain model, and a spatial Markov probability transfer matrix was constructed based on the spatial lag type of each regional unit in the first year. Similarly, according to the quantile division method, the spatial lag types in the study area were divided into four types, namely low, medium-low, medium-high, and high, denoted as types Ⅰ, Ⅱ, Ⅲ and Ⅳ, respectively. The analysis results are listed in Table 4 below. Through comparison with the traditional Markov probability transfer matrix, the following spatial evolution characteristics of the CLUE could be obtained after considering the geospatial background:

Geospatial patterns play a significant role in the dynamic evolution process of the CLUE. Against the neighborhood background entailing different efficiency levels, the CLUE transfer probability varies, and the transfer probability further differs from that determined according to the corresponding traditional Markov probability transfer matrix. For example, in the traditional Markov probability transfer matrix, the transition probability of the CLUE level from type III to type Ⅱ is the highest, at P_III-II_ = 0.385, while in the spatial Markov probability transfer matrix, when a location is adjacent to a type-I area, the transition probability of the CLUE level from type III to type Ⅱ is P_III-II (I)_ = 0.333, which is lower than the probability that the CLUE level remains unchanged. When a given location is adjacent to a type II area, the transition probability is P_III-II (II)_ = 0.400, higher than the probability that the CLUE level remains unchanged. When the adjacent area is a type-IV area, the transition probability is P_III-II (IV)_ = 0.411 = P_III-III (IV)_. The probability that the CLUE level remains unchanged is the same as the transition probability to a medium-low efficiency level. Therefore, geographical spatial patterns can exert a significant impact on CLUE evolution.

The spatial spillover effect plays a vital role in the dynamic transfer process of the CLUE. Generally, the transfer probability of the CLUE to reach a low level increases in areas adjacent to cities with low CLUE values, while the transfer probability of the CLUE to reach a high level increases at locations adjacent to areas with high CLUE values. For example, near areas with low CLUE values, P_II-I (I)_ (0.231) > P_II-I_ (0.190) and P_III-II (I)_ (0.333) > P_III-II_ (0.385), while at locations near areas with high CLUE values, P_I-II (IV)_ (0.125) > P_I-II_ (0.117) and P_II-III (IV)_ (0.111) > P_II-III_ (0.085). Areas with low CLUE values exert a negative impact on surrounding areas, while areas with high CLUE values impose a positive spillover effect on adjacent areas. Adopting Chizhou city, Anhui Province, and Ganzhou city, Jiangxi Province, as examples, from 2008 to 2018, the average CLUE value in the surrounding cities of Chizhou reached only 0.66, which affected the decline in CLUE in Chizhou city from 0.64 to 0.48 to a certain extent. The average CLUE value in the surrounding cities of Ganzhou reached as high as 0.82, which increased the CLUE in Ganzhou from 0.61 to 0.75.

## 4. Discussion

### 4.1. Policy Recommendations

Based on the above research results and analysis conclusions, the construction of National Pilot Zones for Ecological Conservation in China provides a suitable practical foundation and development trend in terms of cultivated land utilization. Through the spatial spillover effect at the municipal scale, CLUE improvement can be achieved in both cities within the National Pilot Zone for Ecological Conservation in China and surrounding areas, its demonstration and leading roles can be fully manifested, and national ecological civilization construction can be promoted. Based on these findings, the following policy recommendations are outlined:

Based on the determined unbalanced development of the CLUE at the provincial level, it is suggested to widely disseminate model construction experience, formulate provincial ecological measures, and realize CLUE enhancement in surrounding areas of National Pilot Zones for Ecological Conservation in China. Anhui and Hunan should learn from the construction experience of ecological civilization pilot areas similar to Jiangxi, Fujian, and Guizhou, including the formulation and implementation of safe utilization schemes of polluted cultivated land, the establishment of a classification list of cultivated land soil environmental quality categories, and the construction of a responsibility assessment system involving four-level cultivated land protection objectives at the provincial, municipal, county and township levels. Hence, policies and measures should be designed according to local conditions to reverse the downward trend of the CLUE to narrow the gap with areas exhibiting high CLUE values.

Based on the mechanism of the spatial adjacency spillover effect at the prefecture-level city scale, it is suggested to fully manifest the positive spillover effect of H-H CLUE agglomeration areas and establish a city linkage model. By improving the breadth and depth of opening-up policies, strengthening agricultural cooperation, resource flow, and personnel exchange processes, promoting coordinated and balanced development of the whole region, and learning from the improvement model of the CLUE in Ganzhou City, Jiangxi Province, under the influence of the positive spillover effect of neighboring cities, low-level areas should be encouraged to overcome their dilemmas.

Based on the downward transfer trend of the CLUE in the evolution process, it is recommended to thoroughly assess the transfer risk from high to low levels, establish a low-level early warning mechanism, and prevent downward transformation of the regional eco-efficiency. A cultivated land ecological utilization evaluation organization shall be established to promptly adjust and optimize the input-output structure of cultivated land, such as the allocation of low-carbon fertilizers and the optimization of the operation time of cultivated land machinery, based on the monitoring of cultivated land carbon emissions sources such as pesticides, chemical fertilizers and agricultural films and the prediction of cultivated land output, so as to ensure synchronous ecological construction in the process of cultivated land utilization.

### 4.2. Research Limitation and Future Research

In this paper, an evaluation index system of the CLUE is constructed, the SBM-undesirable model is adopted to measure the CLUE, and the spatial spillover effect of the CLUE is analyzed. The following two points should be further examined:

The undesired indicators in the eco-efficiency index system of cultivated land use constructed in this paper only consider carbon emissions, and do not consider nonpoint source pollution. In fact, the use of cultivated land can not only produce a large amount of carbon dioxide, but can also produce nonpoint source pollution, such as water and soil pollution attributed to pesticides and fertilizers. However, due to the regional characteristics of the correlation coefficient of nonpoint source pollution measurements, most current studies consider the same coefficient in China. The lack of regional characteristics may affect the accuracy of regional CLUE assessment. Based on this aspect, this paper only considers carbon emissions to ensure the accuracy of the measurements. The nonpoint source pollution emission coefficient based on regional characteristics should be measured in future research. Nonpoint source pollution and carbon emissions could be incorporated into the unexpected output index in the cultivated land use process to measure the CLUE more accurately.

Based on the prefecture-level city scale, this paper studies the temporal and spatial evolution characteristics and spatial spillover effect of the CLUE from the perspective of comparative analysis, thereby choosing 57 prefecture-level cities in Jiangxi, Fujian, Guizhou, Hunan, and Anhui as the research objects. In the future, microscale multilevel research can be carried out at the farm scale. In fact, as the main body of cultivated land use, based on the research scale, farms can better refine the input-output characteristics of cultivated land use, the cultivated land planting behavior of farmers can be elucidated, and the efficiency value can be determined more accurately. This could provide a greater reference value for follow-up optimization of cultivated land use and ecological civilization construction. Moreover, exploring the eco-efficiency of regional cultivated land use at the meso- and microscales could more comprehensively explain the construction effect of cultivated land use in National Pilot Zones for Ecological Conservation in China and provide a theoretical basis for other regions to learn from the model experience gained in these zones.

## 5. Conclusions

Based on the obtained panel data of cultivated land use pertaining to 57 cities in Jiangxi, Fujian, Guizhou, Hunan, and Anhui from 2008 to 2018, this paper adopts the SBM-undesirable model to measure the CLUE, and Moran’s I and Markov chain models are employed to analyze the corresponding temporal and spatial evolution characteristics. The main conclusions are as follows:

In terms of temporal evolution, the CLUE in the whole region is significantly differentiated during the research period, exhibiting the characteristics of one growth, two stable, and two decline stages. Guizhou reveals a prominent growth trend, Jiangxi and Fujian exhibit high and stable fluctuation characteristics, and Hunan and Anhui demonstrate an overall downward trend. These results indicate that the effect of cultivated land use and ecological construction in the provinces within the National Pilot Zone for Ecological Conservation in China is better than that in Hunan and Anhui provinces.

In terms of the spatial pattern, during the research period, the CLUE in the whole region exhibits a positive spatial autocorrelation that increases year by year, and the spatial spillover effect is observed. In addition, local H-H and L-L agglomeration core areas exhibit expansion and transfer phenomena. Within the considered National Pilot Zone for Ecological Conservation in China, the positive spatial spillover effect is very pronounced. However, at present, the driving effect on the surrounding regions is not notable.

In terms of trend transfer, geospatial patterns and the spatial spillover effect play a significant role in CLUE evolution. The transfer probability of the CLUE against the different geographical backgrounds varies, and a high probability of improvement is attained near cities with high CLUE values. Proximity to cities with low CLUE values can inhibit enhancement, i.e., the core convergence phenomenon occurs.

## Figures and Tables

**Table 1:** Evaluation index system of the CLUE.

Variable Type	Variable	Index Meaning
Input index	Cultivated land input	Actual sown area of crops/1000 hm^2^
	Labor input	Number of employees in the primary industry × (agricultural output value/total output value of agriculture, forestry, animal husbandry, and fishery)/10,000
	Pesticide and fertilizer input	Net amount of pesticide and chemical fertilizer application/t
Expected output index	Agricultural output value	Output value of the planting industry/10,000 yuan
	Grain yield	Total grain output/t
Unexpected output index	Net carbon emissions	Difference between the total carbon emissions of mechanical operation and chemical fertilizer and pesticide application and the total carbon absorption of cultivated land/t

**Table 2:** Global Moran’s I of the CLUE in the study area from 2008 to 2018.

Year	Global Moran’s I	Z-Value	*p*-Value
2008	0.136	1.759	0.039
2009	0.1869	2.1414	0.032
2010	0.1493	1.9436	0.029
2011	0.3403	3.9418	0.003
2012	0.2308	2.8181	0.004
2013	0.2362	2.7097	0.003
2014	0.1942	2.3543	0.009
2015	0.2561	3.0089	0.007
2016	0.191	2.3219	0.009
2017	0.2572	2.9803	0.002
2018	0.3234	3.7426	0.001

**Table 3:** Traditional Markov chain probability transition matrix of the CLUE in the study area from 2008 to 2018.

Local Status	Type Ⅰ	Type Ⅱ	Type Ⅲ	Type Ⅳ
	<25%	25–50%	50–75%	>75%
Type Ⅰ	0.832	0.117	0.007	0.044
Type Ⅱ	0.190	0.647	0.085	0.085
Type Ⅲ	0.058	0.385	0.365	0.192
Type Ⅳ	0.018	0.048	0.079	0.855

**Table 4:** Markov chain probability transition matrix of the CLUE in the study area from 2008 to 2018.

Spatial Lag	Local Status	Type Ⅰ	Type Ⅱ	Type Ⅲ	Type Ⅳ
		<25%	25–50%	50–75%	>75%
Type Ⅰ	Ⅰ	0.770	0.148	0.000	0.082
	Ⅱ	0.231	0.513	0.051	0.205
	Ⅲ	0.000	0.333	0.444	0.222
	Ⅳ	0.077	0.077	0.077	0.769
Type Ⅱ	Ⅰ	0.854	0.122	0.000	0.024
	Ⅱ	0.179	0.678	0.143	0.000
	Ⅲ	0.100	0.400	0.300	0.200
	Ⅳ	0.000	0.048	0.065	0.887
Type Ⅲ	Ⅰ	0.926	0.074	0.000	0.000
	Ⅱ	0.175	0.750	0.050	0.025
	Ⅲ	0.063	0.375	0.313	0.25
	Ⅳ	0.016	0.049	0.098	0.836
Type Ⅳ	Ⅰ	0.750	0.125	0.125	0.000
	Ⅱ	0.139	0.639	0.111	0.111
	Ⅲ	0.059	0.411	0.411	0.118
	Ⅳ	0.001	0.050	0.075	0.863

## Data Availability

The data presented in this study are available on request from the corresponding author.
